# Low-Temperature Catalytic Ozonation of Multitype VOCs over Zeolite-Supported Catalysts

**DOI:** 10.3390/ijerph192114515

**Published:** 2022-11-04

**Authors:** Jiaming Shao, Yunchu Zhai, Luyang Zhang, Li Xiang, Fawei Lin

**Affiliations:** 1 State Key Laboratory of Clean Energy Utilization, Zhejiang University, Hangzhou 310027, China; 2 Zhejiang SUPCON Technology Co., Ltd., Hangzhou 310053, China; 3 China Energy Engineering Group, Zhejiang Electric Power Design Institute Co. Ltd., Hangzhou 310012, China; 4 School of Environmental Science and Engineering, Tianjin University and Tianjin Key Lab of Biomass and Wastes Utilization, Tianjin 300072, China

**Keywords:** VOCs, catalytic ozonation, HZSM, low temperature

## Abstract

Volatile organic compounds (VOCs) are an important source of air pollution, harmful to human health and the environment, and important precursors of secondary organic aerosols, O_3_ and photochemical smog. This study focused on the low-temperature catalytic oxidation and degradation of benzene, dichloroethane, methanethiol, methanol and methylamine by ozone. Benzene was used as a model compound, and a molecular sieve was selected as a catalyst carrier to prepare a series of supported active metal catalysts by impregnation. The effects of ozone on the catalytic oxidation of VOCs and catalysts’ activity were studied. Taking benzene as a model compound, low-temperature ozone catalytic oxidation was conducted to explore the influence of the catalyst carrier, the active metal and the precious metal Pt on the catalytic degradation of benzene. The optimal catalyst appeared to be 0.75%Pt–10%Fe/HZSM(200). The catalytic activity and formation of the by-products methylamine, methanethiol, methanol, dichloroethane and benzene over 0.75%Pt–10%Fe/HZSM(200) were investigated. The structure, oxygen vacancy, surface properties and surface acidity of the catalysts were investigated. XRD, TEM, XPS, H_2_-TPR, EPR, CO_2_-TPD, BET, C_6_H_6_-TPD and Py-IR were combined to establish the correlation between the surface properties of the catalysts and the degradation activity.

## 1. Introduction

Volatile organic compounds (VOCs) are important precursors of O_3_, PM2.5, and photochemical smog and can increase the near-ground ozone concentration and seasonal haze weather, greatly contributing to typical air pollution episodes such as fine particulate pollution in winter and ozone pollution in summer [1]. VOCs in the atmosphere photochemically react with NO_x_ to generate secondary pollutants or intermediate substances with strong chemical reactivity, further enhancing O_3_ pollution and photochemical smog that will endanger crops and people’s lives [2].

At present, VOCs control methods include preventive measures to avoid VOCs leakage, such as changing operating conditions, replacing raw materials and replacing equipment. The current elimination technologies can be classified into destruction and recovery methods. The former include high-temperature regenerative combustion, catalytic oxidation, plasma degradation, photocatalytic degradation and biodegradation [3]. The latter include absorption, adsorption and membrane separation [4]. In the presence of VOCs that at a too low concentration to be recovered, destruction methods are the first choice. Regenerative combustion requires a high energy consumption and always generates many toxic byproducts. Catalytic oxidation can completely transform VOCs into H_2_O, CO_x_, HCl and other non-toxic or slightly toxic small molecular substances that are easy to be separated and collected. Catalytic oxidation is advantageous as it is simple and very efficient. Compared with the combustion method, the temperature required by catalytic oxidation can be reduced to below 600 °C to achieve the complete transformation of VOCs and produce less secondary pollutants; therefore, this technique is often used for the removal of VOCs [5]. However, some problems still exist, such as the relatively high reaction temperature required, catalyst deactivation by Cl/S poisoning, and the production of many byproducts [6]. Other destruction technologies have also the drawbacks of requiring high investments and having a low conversion efficiency.

Studies have found that the presence of ozone can further reduce the temperature of catalytic oxidation even to room temperature, which greatly saves energy [7]. In addition, high efficiency can be achieved, and VOCs can be completely converted to ideal products that are less toxic [8]. The catalytic transformation of VOCs mainly depends on the catalyst’s properties. At present, the commonly used catalysts include noble metal catalysts, supported transition metal oxide catalysts, perovskite catalysts, and molecular sieve catalysts. In recent years, transition metal oxide catalysts, such as MnO_x_, Fe_2_O_3_, Co_3_O_4_, and CuO, have been widely studied in the catalytic oxidation of VOCs [9,10]. These catalysts have the characteristics of a multiple crystal phase structure, sintering resistance, large source, flexibility in synthesis, variable valence state, good thermal stability, and low price. Molecular sieves have abundant pore channels, excellent thermal and chemical stability, allow the easy adjustment of the SiO_2_/Al_2_O_3_ ratio, have surface acidity, hydrophobicity, and good catalytic activity [11]. Liu et al. synthesized Cu modified birnessite MnO_2_ catalysts by the redox method and could achieve a toluene conversion efficiency of 95% at a low temperature, ca. 250 °C [12]. Researchers have also begun to create multi-component active centers for the catalytic oxidation of VOCs. By adding other metals, binary active centers formed, improving the catalytic activity. Deng et al. synthesized a CoMnCe ternary catalyst to promote the formation of oxygen vacancies, which exhibited excellent catalytic activity on benzene at low temperature with excellent CO_2_ selectivity, water resistance and high stability [13]. Ding et al. enhanced the hydroxyl density near the active site of Pt through Ni modification (Pt–Ni/ZSM), thus improving the catalytic transformation on formaldehyde, reaching stability for 100 h and 90% formaldehyde conversion at 30 °C [14].

Zhang et al. synthesized MnCoOx catalysts for chlorobenzene (CB) ozonation, attaining 90% conversion with an O_3_/CB ratio of 10.0 at 120 °C [15]. Xiang et al. reported a series of Mn-based single and binary oxide catalysts for dichloromethane (DCM) ozonation, and the hollow urchin-like morphology contributed to the 100% conversion of DCM with an O_3_/DCM ratio of 10.0 at 120 °C [16]. Chen et al. also compared the catalytic ozonation of benzene with that obtained with Mn-based catalysts on different supports. The ZSM support exhibited the desirable activity [17], but the optimization of ZSM-supported catalysts for the catalytic ozonation of benzene has not been extensively investigated. This study firstly intended to investigate the catalytic ozonation of benzene over a series of ZSM-supported metal oxide catalysts to identify the optimal catalysts. Next, the catalysts were characterized, and the relationship between catalytic activity and catalysts’ property are discussed. Finally, the catalytic ozonation of methanol, methanethiol, methylamine, and dichloroethane was evaluated to verify the application potential of this technology with respect to multiple types of VOCs.

## 2. Experimental Section

### 2.1. Catalysst Preparation

All catalysts were synthesized by the impregnated method. To this aim, 1 g of HZSM(27) (SiO_2_/Al_2_O_3_ = 27), HZSM(117) (SiO_2_/Al_2_O_3_ = 117) and HZSM(200) (SiO_2_/Al_2_O_3_ = 200) powder without any pretreatment, purchased from Nankai Catalysts Co. Ltd., was mixed with an absolute ethanol solution of Mn precursors. Only HZSM(200) was used for other metal-supported catalysts. Mn(CH_3_COO)_2_•4H_2_O, Co(NO_3_)_2_•6H_2_O, Cu(NO_3_)_2_•3H_2_O and Fe(NO_3_)_3_•9H_2_O were used as precursors of transitional metals, i.e., Mn, Co, Cu and Fe, respectively. The loading amount of the metal was fixed at 10 wt%. After thoroughly mixing for 1 h at ambient temperature, the mixtures were evaporated in a water bath at 70 °C. Finally, the obtained samples were calcined at 400 °C for 3 h with a ramp rate of 1 °C/min under a static atmosphere after drying in an oven. These catalysts were labelled 10%Mn/HZSM(27), 10%Mn/HZSM(117), 10%Mn/HZSM(200), 10%Co/HZSM(200), 10%Cu/HZSM(200) and 10%Fe/HZSM(200). Hereafter, different amounts of Pt were loaded on 10%Mn/HZSM(200), using 1, 0.75 and 0.5 wt%. H_2_PtCl_6_•6H_2_O as a precursor in combination with Fe(NO_3_)_3_•9H_2_O. Finally, 1%Pt–10%Fe/HZSM(200), 0.75%Pt–10%Fe/HZSM(200) and 0.5%Pt–10%Fe/HZSM(200) were obtained.

### 2.2. Catalytic Characterization

The pore structure parameters and N_2_ adsorption–desorption curve of the catalysts were determined by using a BMD-PS (M) automatic gas adsorption analyzer (Quantachrome). The catalysts were degassed at 300 °C for 2 h before the experiment. The surface area, total pore volume and average pore size of the samples were determined by the Brunauer–Emmett–Teller (BET) method from the N_2_ adsorption–desorption isotherm and the Barrett–Joyner–Halenda (BJH) method, respectively. The crystal structure of the catalysts was analyzed by an X’Pert Pro powder crystal X-ray diffractometer (XRD) produced by the Panako Company in the Netherlands. The copper target (Cu Kα radiation, λ = 0.154 nm) was used. The test conditions were 40 kV tube voltage and 25 mA tube current. Scanning was performed continuously from 10 to 80° at a speed of 10 °C•min^−1^. The fresh catalysts were tested with a Nicolet 380 Fourier-transform infrared spectrometer (FTIR, Thermo) to identify the Lewis and Brönsted acidic sites. The catalysts were heated in a vacuum reactor at a rate of 10 °C/min to 400 °C and then cooled to 25 °C. Then, pyridine vapor was injected into the system for adsorption. The desorption process was conducted by increasing the temperature to 200 °C at a rate of 10 °C/min. The metal dispersion, particle size distribution, morphology and lattice were analyzed by an FEI F20 high-resolution field-emission transmission electron microscope (HRTEM, F20). An Escalab 250Xi X-ray photoelectron spectrometer (XPS, Thermo Fisher, USA) was used to analyze the valence and binding energy of the surface elements. Monochromatized Al Kα rays (10 mA, HV = 1486.6 eV, 15 kV) were used as the source of X-ray excitation, and the spot size was 500 μm. All binding energies were adjusted based on C 1s at 284.8 eV. The electron paramagnetic resonance (EPR) was measured with an EMX PLUS instrument (Bruck, Germany) to identify oxygen vacancies. H_2_-temperature-programmed reduction (H_2_-TPR) and CO_2_-temperature-programmed desorption (CO_2_-TPD) were evaluated on a Chembet TPD analyzer (Quantachrome). We then loaded 50 and 100 mg of each catalyst into the reactor and purged by He at 150 °C to remove the impurities on the catalyst’s surface. After cooling to 50 °C, the atmosphere was changed to 10% H_2_/He and 5% CO_2_/He, respectively, and adsorption was carried out for 1 h. Subsequently, the flow gas was changed to pure He again and stabilized for 1 h. Next, the temperature was increased to 800 °C at a ramp rate of 10 °C/min to obtain the TPR and TPD profiles. The C_6_H_6_-TPD was measured on a FineSorb3010 instrument with the same temperature variation used for CO_2_-TPD.

### 2.3. Activity Measurements

Catalytic ozonation of multi-type VOCs was conducted in a fixed-bed reactor; the detailed instruments are described in our previous works [18,19]. For the experimental study of the concentration gradient of VOC oxidation by ozone, the reaction temperature was set at 120 °C. The amount of catalyst used was 0.025 g, and the flow rate of the reaction gas was maintained at 100 mL/min, corresponding to the gas hourly space velocity (GHSV) of 40,000 h^−1^. The O_2_ concentration was 10%, and the initial concentration of benzene, methanol, methanethiol, methylamine, and dichloroethane was 50, 300, 300, 300, and 50 ppm, respectively. The effluent gas was sampled every 15 min and determined by a GC (gas chromatographer) equipped with two FI detectors. CO/CO_2_ were converted to CH_4_ in a catalytic reaction in the in GC and detected with the FID. The relationship between the conversion rate and CO/CO_2_ selectivity was calculated according to Equations (1)–(4).

VOC conv. = ([VOC]_inlet_ − [VOC]_outlet_)/[VOC] _inlet_ × 100.0% (1)

CO_2_ sel. = [CO_2_]_outlet_/([CO]_outlet_ + [CO_2_]_outlet_) × 100.0% (2)

CO sel. = [CO]_outlet_/([CO]_outlet_ + [CO_2_]_outlet_) × 100.0% (3)

Mineralization rate = [CO_2_]_outlet_/([VOC]_inlet_ × N_C_) × 100.0% (4)

where [VOC]_inlet_ and [VOC]_outlet_ are the initial and residual concentrations of VOCs in ppm, respectively; [CO]_outlet_ and [CO_2_]_outlet_ are the outlet CO and CO_2_ concentrations, in ppm, respectively; N_C_ is the carbon number in the corresponding VOC molecules.

## 3. Results and Discussion

### 3.1. Catalytic Ozonation of C_6_H_6_ over Zeolite-Supported Catalysts

**Figure 1:**
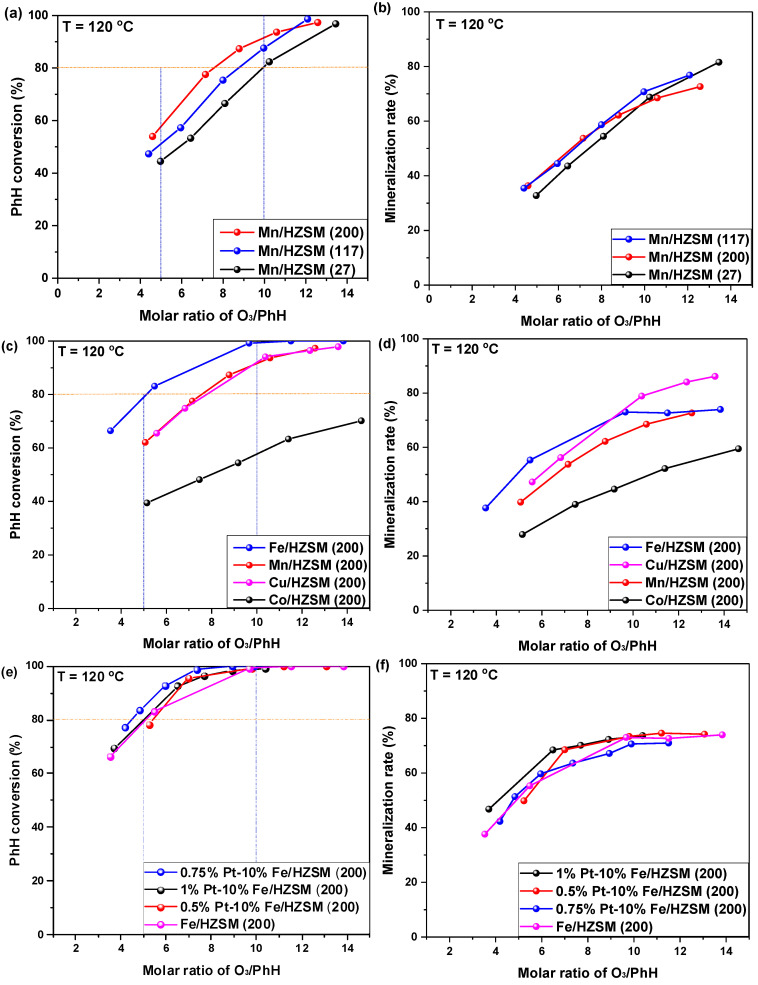
Catalytic ozonation of PhH (C_6_H_6_) over HZSM-supported catalysts. Initial PhH concentration: 100 ppm, O_2_ concentration: 10%, catalyst dosage: 25 mg, reaction temperature: 120 °C, GHSV: 40,000 h^−1^. (**a**,**b**) Catalytic behavior over HZSM supported Mn catalysts with different SiO_2_/Al_2_O_3_ ratios; (**c**,**d**) Catalytic behavior over HZSM(200) supported catalysts with different metals; (**e**,**f**) Catalytic behavior over HZSM(200) supported Fe catalysts with Pt doping.

The catalytic rates of benzene (PhH) conversion and mineralization over Mn-supported HZSM with different Si/Al ratios were investigated and are presented in Figure 1a,b. Clearly, PhH conversion at a O_3_/PhH ratio of 10.0 declined in the following order: Mn/HZSM(200) > Mn/HZSM(117) > Mn/HZSM(27). Generally, a higher Si/Al ratio implies a lower acidity. Therefore, PhH conversion is hindered by the acidity of the catalyst, to some extent. With a O_3_/PhH ratio exceeding 11.0, Mn/HZSM(117) was better than Mn/HZSM(200) and attained the total conversion of PhH at a O_3_/PhH ratio of 12.0. However, Mn/HZSM(200) should also be regarded as an optimal catalyst on the basis of its whole conversion curve. Interestingly, the mineralization rate of these catalysts was very similar for different O_3_/PhH ratios and could reach 60–70% for a ratio of O_3_/PhH of 10.

Hereafter, HZSM(200) was regarded as the optimal support to investigate the effect of different active metals. Figure 1c,d present the corresponding behaviors of 10%Fe/HZSM(200), 10%Mn/HZSM(200), 10%Cu/HZSM(200) and 10%Co/HZ SM-5(200). The difference between the catalysts was much larger than that previously observed. In fact, 10%Fe/HZSM(200) exhibited the highest PhH conversion that could attain 100% at an O_3_/PhH ratio of 10.0. Most importantly, it attained approximately 80% conversion at an O_3_/PhH ratio of 5.0, saving O_3_ consumption significantly. Fe oxides tended to generate a high density of hydroxyl groups, which is favorable for catalytic ozonation, especially for wastewater treatment. In addition, the following characterizations demonstrated that it exhibited a smaller pore size, which increased the PhH adsorption capacity. The PhH conversions obtained with 10%Mn/HZSM(200) and 10%Cu/HZSM(200) were very similar. However, 10%Co/HZSM-5(200) presented a very poor performance. For the mineralization rate, 10%Fe/HZSM(200) exhibited the highest value at an O_3_/PhH ratio lower than 8.5, while 10%Cu/HZSM(200) reached a mineralization of 88% finally. CuO has been reported to be active in CO oxidation at low temperature, which should contribute to its high mineralization rate [20].

To further improve the PhH conversion, the noble metal PtO_x_ was incorporated into 10%Fe/HZSM(200) in different amounts. As presented in Figure 1e,f PtO_x_ loading indeed enhanced the PhH conversion, which increased firstly and then declined with the elevation of Pt loading. We found that 0.75%Pt–10%Fe/HZSM(200) exhibited the highest PhH conversion reaching 98% at an O_3_/PhH ratio of 7.0. However, 1%Pt–10%Fe/HZSM(200) showed the highest mineralization rate at different O_3_/PhH ratios. Herein, 0.75%Pt–10%Fe/HZSM(200) was selected as the optimal catalyst for further investigation.

### 3.2. Textural and Crystal Properties

**Figure 2:**
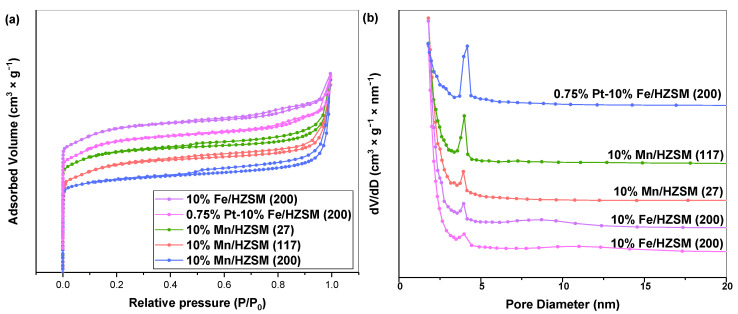
N_2_ adsorption–desorption isotherms (**a**) and pore size distribution (**b**) of the catalysts.

We then investigated the catalytic properties of 10%Mn/HZSM(27), 10%Mn/HZSM(117), 10%Mn/HZSM(200), 10%Fe/HZSM(200) and 0.75%Pt–10%Fe/HZSM(200) during PhH ozonation. A catalyst with a high surface area and a high number of pores can reduce the activation energy of a reaction to a certain extent, which is conducive to the activation of the reactants. The nitrogen sorption–desorption isotherms and pore size distribution curves of the five catalysts are shown in Figure 2a,b. These catalysts exhibited a high absorption rate at a low relative pressure and showed a relatively stable trend under a high pressure, which is a typical feature of zeolite. All catalysts presented type IV isotherms and H3 hysteresis loops, indicating mesoporous structures. Table 1 reports the pore structure parameters of the five catalysts. It shows that 10%Mn/HZSM(200) possessed the largest specific surface area, ca. 374.2 m^2^•g^−1^, while 10% Mn/HZSM(117) exhibited the highest pore volume, ca. 0.25 cm^3^•g^−1^. In addition, 10% Mn/HZSM(200) showed the largest average pore size, 9.3 nm. Metal loading often occupied part of the original pores in the support, leading to a decrease in the specific surface area and pore volume, while the average pore size displayed an uncertain trend. Interestingly, the total pore volume and average pore size of 0.75% Pt–10%Fe/HZSM(200) were 0.13 cm^3^•g^−1^ and 4.8 nm, larger than those of 10%Fe/HZSM(200), indicating a better pore structure. The average pore size of 10%Fe/HZSM(200) and 0.75%Pt–10%Fe/HZSM(200) was stable at approximately 4–5 nm.

**Figure 3:**
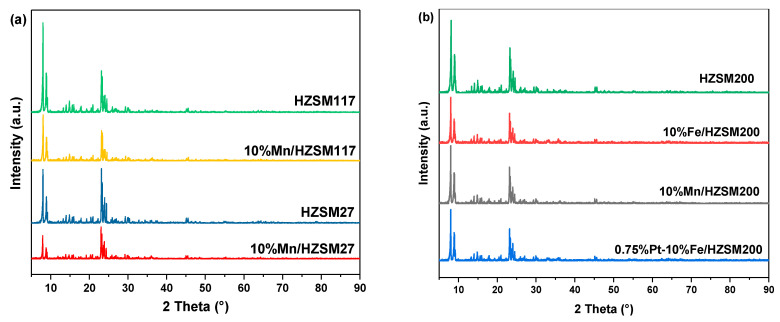
XRD patterns of the catalysts (**a**) HZSM supports and their supported Mn catalysts; (**b**) HZSM200 and its supported catalysts.

The crystalline structure of the catalysts was characterized and analyzed, and the results are shown in Figure 3. Clearly, the diffraction peaks of the 10%Mn/HZSM(27) catalyst basically the same as those of the original HZSM(27) molecular sieve, at 2θ = 8.0°, 8.9°, 23.1°, 23.8°, and 24.4°, indicating that the crystalline structure of HZSM(27) was maintained during the preparation of the catalyst. A similar phenomenon was also observed for HZSM(117) and HZSM(200). Despite further loading of PtO_x_, no obvious changes were detected. In addition, the diffraction peaks corresponding to Mn, Fe, and Pt were not observed, implying a good dispersion of the metals into the supports. The weakening intensity after metal loading indicated declined crystallinity, which generated more defects and active sites for catalytic ozonation.

### 3.3. Surface Physicochemical Analysis

**Figure 4:**
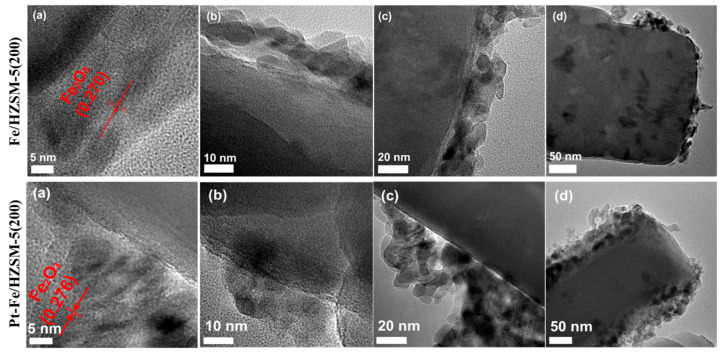
TEM images of 10%Fe/HZSM(200) ((**a**–**d**) in top side) and 0.75%Pt–10%Fe/HZSM(200) ((**a**–**d**) in the bottom side).

The morphology and lattice spacing characteristics of 10%Fe/HZSM(200) and 0.75%Pt–10%Fe/HZSM(200) were studied by TEM, as presented in Figure 4. The lattice spacing of the loaded particles in 10%Fe/HZSM(200) was approximately 0.270 nm, corresponding to the Fe_2_O_3_ crystal plane. For 0.75%Pt–10%Fe/HZSM(200), the lattice spacing of the loaded particles was 0.276 nm, also corresponding to the crystal plane of Fe_2_O_3_. In addition, it can be seen that the particles were mainly distributed on the surface of the support. Generally, when a supported noble metal catalyst is prepared by the traditional impregnation method, due to the limited diffusion of the noble metal precursor in the microporous zeolite molecular sieve, the metal active components tend to accumulate on the zeolite surface, resulting in the formation of large and heterogeneous noble metal nanoparticles.

**Figure 5:**
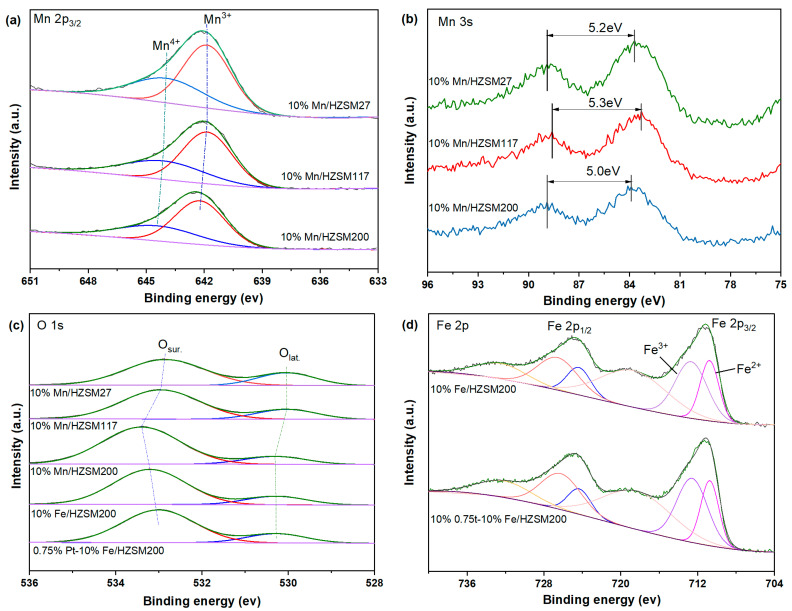
XPS patterns of the catalysts: Mn 2p (**a**), Mn 3s (**b**), O 1s (**c**), and Fe 2p (**d**).

To investigate the valance state of the elemental species on the catalyst surface, the XPS spectra of five fresh catalysts, i.e., 10%Mn/HZSM(27), 10%Mn/HZSM(117), 10%Mn/HZSM(200), 10%Fe/HZSM(200), and 0.75%Pt–10% Fe/HZSM(200) were tested. The bands in the Mn 2p_3/2_ spectra in Figure 5a were deconvoluted into two Gaussian components, corresponding to Mn^3+^ and Mn^4+^ from low to high binding energy [21,22]. Table 2 reports the binding energy position of each peak and the proportion of the Mn valence states. According to previous reports, the content of Mn^3+^ corresponds to the relative amount of oxygen vacancies on the surface. Mn^3+^ is more conducive to ozone oxidation, and Mn^4+^ is more conducive to the deep oxidation of VOCs [19]. The proportion of Mn^3+^/Mn in the 10%Mn/HZSM(200) catalyst was the largest, ca. 64.8%. Therefore, the interaction between Mn and HZSM(200) created more oxygen vacancies, contributing to the highest activity of 10%Mn/HZSM(200), compared to those of the other Mn-supported catalysts, in the catalytic degradation of benzene shown in Figure 1. The Mn 3s spectra are also presented in Figure 5b. The gap between two peaks (ΔE_s_) can be used to calculate the average oxidation state (AOS) of Mn species (AOS = 8.956-1.126ΔE_s_) [23]. In comparison, 10%Mn/HZSM(200) possessed the lowest AOS, corresponding to the highest proportion of Mn^3+^ and the most abundant oxygen vacancies.

The O 1s spectra of the five catalysts shown in Figure 5c were also divided into two characteristic peaks after deconvolution by Gaussian functions. The corresponding binding energy and oxygen species ratios are listed in Table 3. The lattice oxygen (O_lat_.) and surface adsorbed oxygen (O_sur_.) peaks were located at approximately 530.0 and 533.0 eV, respectively. Previous studies have found that the existence of O_sur_. with high mobility is conducive to electron transfer, thus promoting the entrance of oxygen molecules into oxygen vacancies [24,25]. Therefore, O_sur_. plays an important role in catalytic ozonation. Clearly, the proportion of O_sur_. for the 10%Mn/HZSM(200) catalyst was the highest among the Mn-supported catalysts, which also corresponded to its high catalytic activity. In comparison, 10%Fe/HZSM(200) possessed a relatively lower ratio of O_sur_., ca. 83.1%, and a further loading of PtO_x_ decreased the value to 81.5%. To some extent, the intrinsic properties of Fe and Pt oxides played a more important role in catalytic ozonation.

The Fe 2p spectra shown in Figure 5d also contained two characteristic peaks located at 724.7 and 711.2 eV, corresponding to Fe 2p_1/2_ and Fe 2p_3/2_, respectively [26]. Next, they were divided into two characteristic peaks after deconvolution by Gaussian functions, and their corresponding binding energy and the proportion of Fe species are reported in Table 4. Fe^2+^ and Fe^3+^ can be distinguished in the region of Fe 2p_3/2_ from a low to a high binding energy [27]. Another peak located at a higher binding energy was ascribed to satellite Fe species. The coexistence of Fe^2+^ and Fe^3+^ plays an important role in the catalytic oxidation of ozone at low temperature, contributing to the good catalytic oxidation performance of Fe-supported catalysts observed with benzene. Clearly, a further loading of PtO_x_ increased the proportion of Fe^3+^ from 61.5% to 62.6%, which enhanced the redox ability.

**Figure 6:**
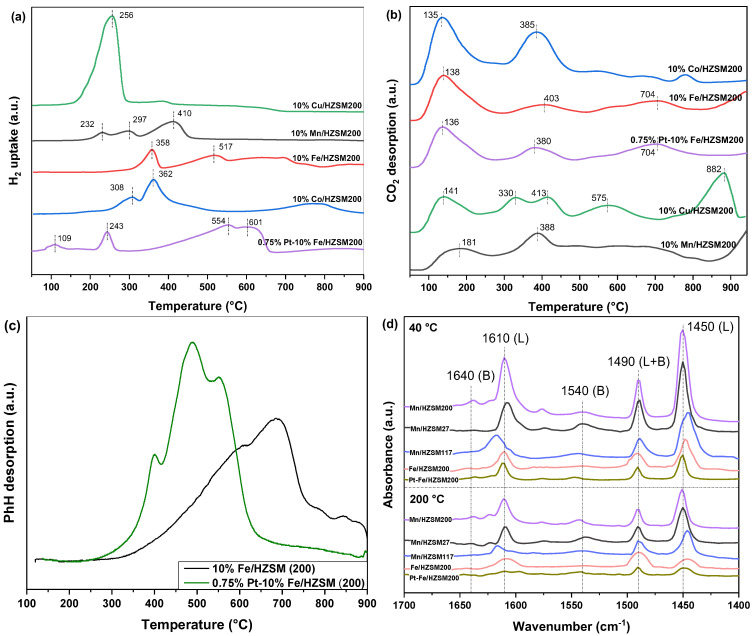
H_2_−TPR (**a**), CO_2_−TPD (**b**), C_6_H_6_−TPD (**c**), and Py−IR (**d**) profiles of the catalysts.

In order to study the redox capacity of the five catalysts, the catalysts with different metal loadings were characterized by H_2_−TPR, and the results are shown in Figure 6a. Most obviously, 10%Cu/HZSM(200) presented only one peak at 256 °C, which was the strongest reduction peak observed when considering all catalysts. Generally, CuO reduction occurred at a lower temperature compared with other metal oxides, thus contributing to the optimal reductive properties of this catalyst [28]. We found that 10%Mn/HZSM(200) showed three reduction peaks, corresponding to the sequential reduction of MnO_2_(IV) to Mn_2_O_3_(III), Mn_3_O_4_(II and III), and MnO(II) [16]. The last reduction peak was the strongest one, which was also consistent with the higher content of Mn^3+^ compared to Mn^4+^. We observed that 10%Fe/HZSM(200) possessed two reduction peaks; the first one corresponded to the reduction of Fe_2_O_3_ to Fe_3_O_4_ and the partial reduction of Fe_3_O_4_ to FeO, and the second one should originate from the reduction of Fe_3_O_4_ and FeO to Fe. However, the second peak was spread over a wide range of temperatures, indicating that the reduction of Fe_3_O_4_ and FeO was difficult. We found that 10%Co/HZSM(200) mainly exhibited two reduction peaks, the first one corresponding to the reduction of Co_3_O_4_ to CoO and in part of CoO to Co, and the second one corresponding to the reduction of Co^2+^ to Co [15]. Most interestingly, the reduction peaks of 0.75%Pt–10%Fe/HZSM(200) were stronger than those of 10%Fe/HZSM(200). Firstly, a new reduction peak emerged at 109 °C. Secondly, the second reduction peak located at 243 °C was also much lower than at that at 358 °C. Finally, the last reduction peaks showing two overlapping peaks at 554 and 601 °C became particularly large. These phenomena indicate significantly improved reduction properties, which should originate from the PtO_x_ incorporation and the strong interactions between Pt and Fe, as well as with the supports. Table 5 lists the quantitative amounts of H_2_ uptake in the H_2_−TPR profiles, which were calculated by integration and normalized by CuO reduction. Obviously, among the five catalysts, 10%Cu/HZSM(200) exhibited the highest H_2_ uptake, ca. 0.81 mmol g-cat^−1^. However, Cu was not the optimal metal for catalytic oxidation and ozone decomposition. Meanwhile, 10%Cu/HZSM(200) possessed excellent redox properties but undesirable catalytic activity. By contrast, 10%Fe/HZSM(200) possessed a lower H_2_ uptake, but exhibited a good PhH conversion performance. Therefore, the metal itself is the most important factor affecting a catalyst’s performance compared with other factors. The coexistence of Pt and Fe indeed enhanced the redox properties and contributed to a high catalytic performance. Pt, a noble metal, has a validated superiority in catalytic oxidation due to its excellent reducibility [29]. Therefore, a trace loading of PtO_x_ is a good choice to improve a catalyst’s performance.

The adsorption of reactants and the desorption of products are important steps in catalytic reactions. CO_2_ is one of the main reaction products of VOCs catalytic degradation. Therefore, the CO_2_ desorption properties are critical for the regeneration of a catalyst’s active sites. Figure 6b shows the CO_2_–TPD curves of the five catalysts. CO_2_ desorption mainly occurred at a temperature lower than 400 °C, except for 10%Cu/HZSM(200). In comparison, 10%Fe/HZSM(200) exhibited stronger CO_2_ desorption peaks than 10%Mn/HZSM(200). Further, the PtO_x_ loading declined the peak temperature. Therefore, the PtO_x_ loading further improved the CO_2_ desorption, thus contributing to an optimal catalytic performance.

In order to understand the adsorption and desorption ability of critical reactants, e.g., PhH, PhH–TPD was characterized using the two optimal catalysts, as shown in Figure 6c. Clearly, PhH desorption initiated at 250 °C and was not complete until 900 °C. Interestingly, only 0.75% Pt loading significantly improved PhH adsorption capacity. The starting temperature for 0.75%Pt–10%Fe/HZSM(200) was much lower than that for 10%Fe/HZSM(200), ca. 250 < 300 °C. Meanwhile, PtO_x_ loading also affected the temperature. Therefore, although these two catalysts possessed similar total PhH adsorption capacity, PtO_x_ loading obviously improved PhH adsorption and desorption at a low temperature, which led to the activation and deep oxidation of PhH during catalytic ozonation.

To evaluate the acid site distribution in the catalysts, pyridine infrared (Py–IR) spectra were collected at 40 and 200 °C, as shown in Figure 6d. Typically, the bands at 1450 and 1600 cm^−1^ corresponded to Lewis acid sites, and the bands at 1540 and 1640 cm^−1^ were ascribed to Brönsted acid sites. Another band at approximately 1490 cm^−1^ originated from pyridine adsorbed at both Brönsted and Lewis sites [30]. Clearly, both Brönsted and Lewis acid sites existed in all these catalysts, but Lewis sites were more abundant. When the temperature rose from 40 to 200 °C, the strength of the Lewis acid sites decreased rapidly, indicating that they were medium-strong acids. Py−IR spectra were used to quantitatively analyze the acid site types of these catalysts, and the results are tabulated in Table 6. It has been reported that Lewis acid sites are the core active sites for C—C cleavage; therefore, 0.75%Pt–10%Fe/HZSM(200) with the highest Lewis acidity was expected to exhibit the optimal catalytic behavior. The Lewis acid concentration of 0.75%Pt–10%Fe/HZSM(200) increased from 30.4 to 50.8 μmol•g^−1^ at 200 °C after Pt loading. Although it was lower than that of 10%Mn/HZSM(200), ca. 57.7 μmol•g^−1^, it was much higher at 40 °C, ca. 174.8 μmol•g^−1^. Coordinated unsaturated Pt species are capable of pyridine adsorption due to their ability to receive electrons; therefore, highly dispersed Pt species may also generate new Lewis acid sites.

**Figure 7:**
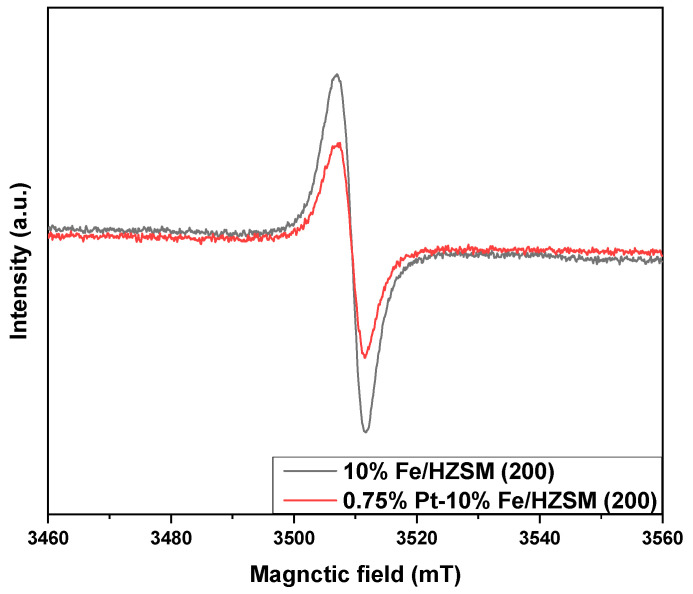
EPR curves of the catalysts.

Figure 7 shows the EPR curves of 0.75%Pt–10% Fe/HZSM(200) and 10%Fe/HZSM(200) to compare the oxygen vacancy. Each sample showed a symmetric EPR signal peak at g = 2.004, which was attributed to unpaired electrons in the oxygen vacancies of molecular sieves; the signal intensity can reflect the concentration of the oxygen vacancies. Clearly, 10%Fe/HZSM(200) exhibited the strongest signal, indicating that there were more oxygen vacancies in the internal lattice of 10%Fe/HZSM(200). The lower EPR signal obtained for 0.75%Pt–10% Fe/HZSM(200) could be caused by the occupation of a part of the oxygen vacancies by PtO_x_. This is also consistent with the XPS results of O 1s for 0.75%Pt–10% Fe/HZSM(200) and 10%Fe/HZSM(200).

### 3.4. Catalytic Ozonation of Multitype VOCs Using the Optimal Catalyst

We found that 0.75%Pt–10%Fe/HZSM(200) achieved good results in the catalytic oxidation of benzene by ozone at a low temperature. However, the composition of actual flue gases is very complex and includes various types of VOCs. Hence, we explored the catalytic oxidation of different VOCs by the catalysts. Typical VOCs containing oxygen, sulfur, nitrogen, and chlorine were selected for the investigation, i.e., methanol (MeOH), methanethiol (MTHM), methylamine (MMA) and dichloroethane (DCE).

**Figure 8:**
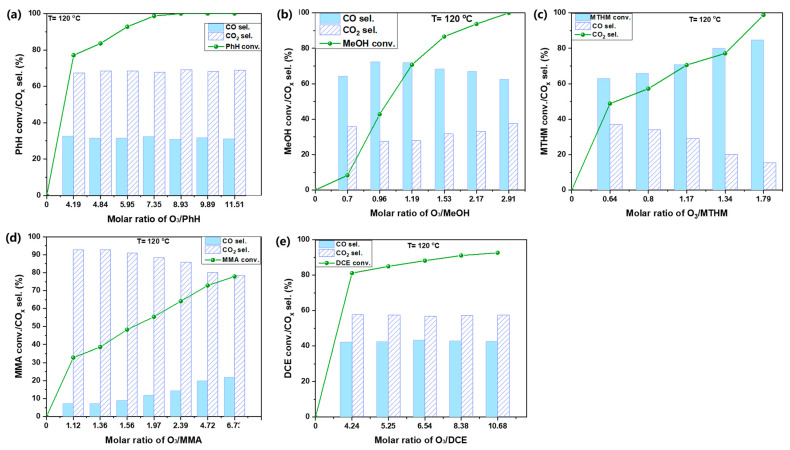
Catalytic ozonation of multitype VOCs with the optimal catalyst (**a**) PhH; (**b**) MeOH; (**c**) MTHM; (**d**) MMA; and (**e**) DCE.

Figure 8 presents the catalytic transformation of different VOCs over 0.75%Pt–10%Fe/HZSM(200) as a function of the O_3_/VOCs ratio at 120 °C. PhH conversion reached 100% when the O_3_/PhH ratio was 8.93; CO and CO_2_ generation remained unchanged along as the O_3_/PhH ratio varied. CO_2_ was dominant, ca. ~70%, compared with CO. Because of the lower carbon number of MeOH compared to PhH, it could be degraded effectively with a low O_3_ input. Therefore, the initial concentration of MeOH was set to 300 ppm. MeOH conversion could attain 100% with the O_3_/MeOH ratio of 2.91. By contrast, CO was dominant and varied significantly as a function of O_3_/MeOH, ca. 60~70%. MTHM possesses a similar molecular structure as MeOH, but O, present in MeOH, is substituted by S. In comparison, the S-H bond is easier to be broken than the O-H bond. Therefore, MTHM could be oxidized at a lower O_3_ input than MeOH, and 100% conversion of MTHM was attained at an O_3_/MTHM ratio of 1.79 and an initial concentration of 300 ppm. CO selectivity was still higher than CO_2_ selectivity and increased continuously with the elevation of the O_3_/MTHM ratio, finally reaching 80%. In MMA, an amino group (NH_2_-) substitutes CH_4_. Two N-H bonds should be harder to be degraded than an O-H and an S-H bond. Accordingly, MMA only attained 80% conversion at an O_3_/MMA ratio of 6.73. However, CO_2_ selectivity was much higher than that for the others and reached 90%. Next, DCE with two C-Cl bonds was more difficult to be converted. Although DCE conversion reached 80% when the O_3_/DCE ratio was 4.24, it increased slowly with further elevation of the O_3_/DCE ratio. CO/CO_2_ selectivity also maintained a negligible variation as the O_3_/DCE ratio varied, which was similar to what observed with PhH. The difficulty in the degradation varied following this order, considering the required O_3_/VOCs for a certain degree of conversion: MMA > DCE > PhH > MeOH > MTHM. MTHM containing S was the easisest to oxidize by the catalytic ozonation method.

### 3.5. Products Distribution and Surface Properties of the Spent Catalysts

Due to the presence of S, N and Cl, additional products were also generated during catalytic ozonation, besides CO_x_ and H_2_O. To detect these products, the effluent gas was absorbed by a 0.0125 M NaOH solution for 150 min, and the ions were detected by an IC instrument. Equations (5)–(10) present the reactions between these products and NaOH. Table 7 reports the detected ions. Large amounts of sulfates and sulfites were generated, indicating SO_2_ formation during the catalytic ozonation of MTHM. Due to its strong oxidizing properties, SO_2_ should be the dominant product containing S. Therefore, the catalytic ozonation of MTHM combined with wet absorption can effectively eliminate MTHM without the formation of secondary pollutants. Similarly, nitrates and nitrites were detected in the effluent gas of NMA, indicating the co-existence of NO and NO_2_. Only Cl^−^, but not ClO^−^, was detected for DCE. That meant that HCl was the only product containing Cl during the catalytic ozonation of DCE. Generally, Cl_2_ is hard to be eliminated in the industry. Hence, HCl formation is expected and favorable for a post-treatment.

Cl_2_ + NaOH → NaCl + NaClO + H_2_O (5)

HCl + NaOH → NaCl + H_2_O (6)

2NaOH + SO_2_ → Na_2_SO_3_ + H_2_O (7)

2NaOH + SO_3_ → Na_2_SO_4_ + H_2_O (8)

2NO_2_ + 2NaOH → NaNO_3_ + NaNO_2_ + H_2_O (9)

NO_2_ + NO + 2NaOH → 2NaNO_2_ + H_2_O (10)

## 4. Conclusions

This study focused on the catalytic degradation of various VOCs, i.e., benzene, methanol, methanethiol, methylamine and dichloroethane, over zeolite-supported catalysts by ozone at a low temperature. The effects of the ozone input on the catalytic conversion of the VOCs were studied. Mn/HZSM(200) showed the highest conversion efficiency for PhH, while the conversion efficiency of other catalysts decreased in the following order: 10%Mn/HZSM(200) > 10%Mn/HZSM(117) > 10% Mn/HZSM(27). A higher ratio of SiO_2_/Al_2_O_3_ corresponded to a better catalytic degradation of benzene. We found that 10%Mn/HZSM(200) possessed an excellent pore structure, the most abundant surface oxygen vacancies, the highest redox capacity, and a high surface acidity. These excellent properties effectively promoted ozone decomposition and the adsorption of PhH molecules as well as its transformation, contributing to the highest benzene conversion among those observed for the studied zeolite-supported Mn catalysts. Next, Fe, Mn, Cu, and Co supported on HZSM(200) were investigated in the catalytic ozonation of benzene. We observed that 10%Fe/HZSM(200) achieved the highest benzene conversion; the conversion efficiency of the other catalysts decreased in the following order: 10%Mn/ HZSM(200) > 10%Cu/HZSM(200) > 10%Co/HZSM(200). PtO_x_ was further loaded on 10%Fe/HZSM(200) to enhance benzene conversion. We found that a loading amount of 0.75% was the optimal one. The improved CO_2_ desorption property, surface acidity, and benzene adsorption capacity were verified by CO_2_-TPD, Py-IR, and PhH-TPD. The catalytic activity and by-product formation over 0.75%Pt–10% Fe/HZSM(200) during the catalytic ozonation of benzene, methanol, methanethiol, methylamine and dichloroethane were investigated. The degradation difficulty followed this order based on the O_3_/VOCs ratio required for the same conversion: MMA > DCE > PhH > MeOH > MTHM. By analyzing the by-products, the S-containing products from the catalytic ozonation of methanethiol mainly contained SO_2_, dichloroethane only contained HCl without Cl_2_, and methylamine contained NO and NO_2_. In conclusion, catalytic ozonation is a promising approach for the elimination of multi-type VOCs at a low temperature.

## Figures and Tables

**Table 1:** Textural properties of the catalysts.

Catalysts	BET Sur. Area/m^2^·g^−1^	Total Pore Vol. ^a^/cm^3^·g^−1^	Aver. Pore Dia. ^b^/nm
10%Mn/HZSM(27)	369.4	0.13	6.1
10%Mn/HZSM(117)	361.9	0.25	2.8
10%Mn/HZSM(200)	374.2	0.15	9.3
10%Fe/HZSM(200)	373.5	0.12	4.3
0.75%Pt–10%Fe/HZSM(200)	372.6	0.13	4.8

^a^ BJH desorption cumulative pore volume. ^b^ BJH desorption average pore diameter.

**Table 2:** Binding energy and proportion of each Mn species.

Catalysts	Mn^4+^		Mn^3+^	
	B.E. (eV)	Mn^4+^/Mn (%)	B.E. (eV)	Mn^3+^/Mn (%)
10%Mn/HZSM(27)	643.9	36.7	641.9	63.3
10%Mn/HZSM(117)	644.1	36.8	641.8	63.2
10%Mn/HZSM(200)	644.4	35.2	642.2	64.8

**Table 3:** Binding energy and proportion of each O species.

Catalysts	O_lat_.		O_sur_.	
	B.E. (eV)	O_lat_./O (%)	B.E. (eV)	O_sur_./O (%)
10%Mn/HZSM(27)	530.0	26.0	532.9	74.0
10%Mn/HZSM(117)	530.0	20.6	533.0	79.4
10%Mn/HZSM(200)	530.3	14.5	533.4	85.5
10%Fe/HZSM(200)	530.3	16.9	533.2	83.1
0.75%Pt–10%Fe/HZSM(200)	530.3	18.5	533.0	81.5

**Table 4:** Binding energy and proportion of each Fe species.

Catalysts	Fe^2+^		Fe^3+^	
	B.E. (eV)	Fe^2+^/Fe (%)	B.E. (eV)	Fe^3+^/ Fe (%)
10%Fe/HZSM(200)	710.8	38.5	712.7	61.5
0.75%Pt–10%Fe/HZSM(200)	710.7	37.4	712.6	62.6

**Table 5:** H_2_ uptake from the H_2_−TPR profiles of the different catalysts.

Catalysts	Fir. Red. Peak	Sec. Red. Peak	Thir. Red. Peak	H_2_ Uptake
	°C	°C	°C	mmol g-_cat_^−1^
10%Mn/HZSM(200)	256	--	--	0.33
10% Fe /HZSM(200)	232	297	410	0.44
10%Co/HZSM(200)	358	517	--	0.58
10%Cu/HZSM(200)	308	362	--	0.81
0.75%Pt–10%Fe/HZSM(200)	243	554	601	0.62

**Table 6:** Quantitative analysis of Brönsted and Lewis acids from the Py-IR spectra.

Catalysts	Temperature/°C	B Acid/μmol·g^−1^	L Acid/μmol·g^−1^	B/L
10%Mn/HZSM(27)	40.0	44.9	178.2	0.25
	200.0	29.5	86.7	0.34
10%Mn/HZSM(117)	40.0	36.1	157.2	0.23
	200.0	19.5	65.4	0.30
10%Mn/HZSM(200)	40.0	14.1	97.7	0.14
	200.0	9.5	57.7	0.16
10%Fe/HZSM(200)	40.0	9.0	81.8	0.11
	200.0	6.5	30.4	0.21
0.75%Pt–10%Fe/HZSM(200)	40.0	13.0	174.8	0.074
	200.0	8.8	50.8	0.17

**Table 7:** IC measurement results of the outlet gas adsorbed by a NaOH solution.

VOCs	$ClO^{-}$	$Cl^{-}$	$SO_{4}^{2-}$	$SO_{3}^{2-}$	$NO_{3}^{-}$	$NO_{2}^{-}$
MTHM	--	--	392.52	251.98	--	--
MMA	--	--	--	--	13.18	23.68
DCE	--	87.40	--	--	--	--
